# Incidence of venous thromboembolism and association with PD-L1 expression in advanced non-small cell lung cancer patients treated with first-line chemo-immunotherapy

**DOI:** 10.3389/fonc.2023.1221106

**Published:** 2024-01-08

**Authors:** Liliana Aguiar De Azevedo, Charles Orione, Cécile Tromeur, Francis Couturaud, Renaud Descourt, Margaux Geier

**Affiliations:** ^1^ Service de Pneumologie, Centre Hospitalier Regional Universitaire de Brest, Brest, Brittany, France; ^2^ Service de Pneumologie, Centre Hospitalier de Cornouaille, Quimper, France; ^3^ Service d’oncologie médicale, Centre Hospitalier Regional Universitaire de Brest, Brest, Brittany, France

**Keywords:** venous thromboembolism, non-small cell lung cancer, PD-L1 expression, chemo-immunotherapy, VTE recurrence, bleeding complications

## Abstract

**Background:**

Venous thromboembolism (VTE) is a serious complication in non-small cell lung cancer (NSCLC) patients. The use of thromboprophylactic therapy is subject to an accurate assessment of the VTE risk depending on patients, tumor characteristics and type of systemic antineoplastic treatments. However, little is known concerning the risk of VTE in patients suffering from an advanced NSCLC treated with first-line chemo-immunotherapy and the impact of tumor biomarkers such as PD-L1 expression.

**Methods:**

We performed a retrospective, observational, single-centre study in a cohort of advanced NSCLC patients treated with first-line chemo-immunotherapy. The primary endpoint was the incidence of VTE. Secondary endpoints were the cumulative incidence of VTE, the impact of PD-L1 on VTE occurrence, overall survival, the rate of VTE recurrence under anticoagulant treatment and the rate of bleeding complications.

**Results:**

109 patients were included, of whom 21 (19.3%) presented a VTE event during a median follow-up of 13 months. VTE incidence at 3, 6 and 12 months was 12.1%, 15.1% and 17.5% respectively. 61% were pulmonary embolisms, 9.5% were isolated deep vein thrombosis and 14.3% were central venous catheter-related thrombosis. Our study did not show a significant impact of PD-L1 on VTE occurrence. Overall survival at 6, 12 and 24 months was 81.9%, 74.4% and 70.3% respectively. Four patients developed a recurrent VTE under anticoagulation therapy 3 to 5 months after the first VTE event. One patient suffered from a major bleeding complication while under anticoagulation therapy, leading to death.

**Conclusion:**

VTE is a common complication in advanced NSCLC patients treated with concomitant chemo-immunotherapy. In our study, 19.3% of patients developed a VTE during a median follow-up of 13 months. PD-L1 did not appear to be associated with VTE occurrence. We recorded high VTE recurrence rates despite anticoagulant treatment. Further investigations are needed to determine if high PD-L1 expression is associated with VTE.

## 1 Introduction

Cancer is one of the major known risk factors of venous thromboembolism (VTE) (1, 2) and one of the main causes of mortality in patients developing VTE (3). On the other hand, VTE is correlated with a lower survival and represents the second leading cause of death in cancer patients (4). Moreover, VTE recurrences are more frequent in cancer patients than in general population (5–7) and have higher mortality rates, especially in lung cancer patients (8).

Cancer-associated VTE risk varies according to cancer type, the presence of metastases or disease progression, but also the use of systemic treatments such as chemotherapy (9). VTE risk scores have been developed, such as the Khorana score (4), to help identify patients at high risk that might benefit from a thromboprophylactic treatment. According to current guidelines, direct oral anticoagulants (DOACs) can be used as an alternative to low-molecular weight heparin (LMWH) and must be preferred to vitamin K antagonists (VKAs) in treatment or prophylaxis of cancer-associated VTE (10, 11). Indeed, four randomized controlled trials have shown that DOACs are as efficient and as safe as LMWH in cancer-associated VTE (12–15). However, the high rates of bleeding complications in cancer patients under anticoagulation make it necessary to carefully assess the VTE risk factors and the indication for anticoagulant treatment in this population (7, 16).

Lung cancer is one of the most common cancers and is the leading cause of cancer-related mortality in France and worldwide. Lung cancer has one of the highest incidence rates of VTE − ranging from 3% to more than 20% according to several studies (17–19) − as well as one of the highest rates of VTE recurrence and bleeding complications among solid cancers (7, 16). Among non-small cell lung cancer (NSCLC), the subtype adenocarcinoma is associated with a higher risk of VTE (20, 21) and this risk might be increased by chemotherapy and other systemic treatments such as molecules interfering with the vascular endothelial growth factor (VEGF) pathway (9).

In recent years, the advent of immunotherapy has revolutionized the treatment and prognosis of NSCLC. The first-line current standard of care of advanced NSCLC without actionable driver mutations is based on the association of a platinum-based chemotherapy and immunotherapy (22, 23). If chemotherapy or anti-VEGF are widely recognized risk factors of VTE, the risk associated with immune checkpoint inhibitors remains unclear (9, 24, 25). Tumor molecular and genetic characteristics also appear to play a role in VTE formation (26). Several studies have shown an association between the existence of *ALK* or *ROS1* translocations and the occurrence of VTE in NSCLC (26–29). This association is less clear concerning the impact of other tumoral markers: some studies suggest a higher VTE risk in patients with *KRAS* mutations and no association or a protective effect of *EGFR* mutations (26, 29–32). Moreover, little is known about the impact of the programmed death-ligand 1 (PD-L1) expression regarding the risk of VTE. PD-L1 is a protein expressed at the surface of tumor cells that inhibits the anti-tumor immune response. Immunotherapy drugs target PD-L1 or its receptor on T cells (PD-1) and hence interrupt the inhibition signal. PD-L1 score is a predictive marker of the efficacy of immunotherapy (22, 23). Therefore, immunotherapy and the level of PD-L1 expression could play an important role in the coagulation cascade and in the thrombosis mechanisms by activating the immune response, by generating an inflammatory tumor microenvironment and by enhancing the tissue factor expression in activated immune cells (33–36). Several studies suggest an association between the expression of PD-L1 and the incidence of VTE in NSCLC (25–27, 37). Therefore, in our study, we aimed at determining VTE incidence in NSCLC patients treated with the association of chemotherapy and immunotherapy (CT-IO) in first-line and to assess if PD-L1 is an independent risk factor of VTE.

## 2 Methods

### 2.1 Study population and design

We performed an observational retrospective single-centre cohort study at the Brest University Hospital. Patients were first identified by using the CHIMIO^®^ software database. Patients were included if they were 18 years of age or older; if they had a confirmed diagnosis of an advanced stage NSCLC (metastatic, locally advanced non eligible for surgery or radiotherapy; or metastatic relapse of an initially localized tumor); if they had received at least one dose of a first-line treatment with an association of CT-IO according to current standards of care (a platinum-based chemotherapy associated either to pemetrexed for the non-squamous subtypes or to paclitaxel for the squamous subtype (SCC) with an inhibitor of PD-1: pembrolizumab); and if they had given their non-opposition agreement to participate in the study.

Exclusion criteria were: a small-cell histology; a previous systemic therapy for metastatic disease; patients presenting a synchronous diagnosis of cancer; patients followed in an outside institution; patients under legal protection measures and patients refusing to participate.

All patient’s relevant clinical and biological data were collected from the electronic medical record as well as tumor profile and successive lines of cancer treatment. VTE events included pulmonary embolism (PE) associated or not to deep vein thrombosis (DVT), DVT alone, central venous catheter-related thrombosis (CRT) and extensive superficial vein thrombosis (SVT) requiring an anticoagulant treatment, other vein thrombosis (superior vena cava, jugular and upper limbs veins or visceral vein thrombosis) occurring in the three months before the cancer diagnosis (inaugural VTE), during CT-IO treatment or after the first-line treatment. All VTE events were confirmed by an imaging exam: computed tomography pulmonary angiography (CTPA) or ventilation/perfusion scan for PE, doppler ultrasound for DVT, or angiography for CRT or others. Incidental VTE events were also considered. VTE recurrence was defined as a venous thrombosis in a new site or an extension of the thrombosis at the initial site. Use of anticoagulant treatment and bleeding complications were reviewed. Bleeding complications were classified according to the International Society on Thrombosis and Haemostasis (ISTH) definitions in major and minor bleeding.

### 2.2 Study outcomes

The primary endpoint was to determine the incidence of VTE in our cohort (i.e. VTE occurring in the three months before the cancer diagnosis (inaugural VTE), during CT-IO treatment or after the first-line treatment).

Secondary endpoints included:

1. The cumulative incidence of VTE from date of cancer diagnosis at specific time of interest (3, 6 and 12 months)
2. VTE occurrence according to PD-L1 expression (divided in three subgroups: PD-L1 < 1%; PD-L1: 1-49% and PD-L1 ≥ 50%).
3. Overall survival (OS) measured from start of first-line CT-IO treatment to death or censured at date of data collection for alive patients
4. VTE recurrence rate and characteristics
5. Bleeding complications under anticoagulation treatment

### 2.3 Statistical analysis

Descriptive analyses were done using median (± interquartile range [IQR]) for quantitative variables, and percentages for categorical variables. Comparison between the VTE group and the no-VTE group were performed using Student’s *t*-test or Wilcoxon nonparametric test. Chi-square or Fisher’s tests were used for categorical variables. A *p*-value < 0.05 was considered as statistical significance. VTE-free survival and overall survival were estimated by the Kaplan-Meier method. Estimations of cumulative incidence and 95% confidence intervals (95% CI) were calculated. All statistical analysis were performed using the RStudio^®^ software (RStudio Desktop 2022.02.3 + 492 version).

### 2.4 Ethical considerations

This non-interventional study was approved by the Institutional Review Board of the French learned society for respiratory medicine – “ Société de Pneumologie de Langue Française” – (reference CEPRO 2022-041), by a regional ethics committee and France’s national data protection authority (CNIL), according to French law. All patients alive received written information before enrolment. Opposition should be expressed within 15 days in case of refusal.

## 3 Results

### 3.1 Patient’s baseline characteristics

From December, 2019 to February, 2022, a total of 112 patients received a first line treatment with CT-IO for an advanced NSCLC at the Department of Oncology of the Brest University Hospital. Three of them were excluded because of a synchronous neoplasm. A total of 109 patients were included in the analyses.

Population characteristics were well balanced between patients who developed VTE (VTE group n=21) and those who did not (no-VTE group n=88) (**Table 1**). Median age of patients was 62.7 years (37 to 79 years); 37.9% were females; 98.1% were current or former smokers; the main comorbidities were chronic respiratory and cardiovascular diseases. Only one patient (in the no-VTE group) had a prior VTE history. In the VTE group, 19.0% of patients had a prior cancer history compared to 11.4% in the no-VTE group but this difference was not statistically significant. The rate of immobility was significantly higher in the VTE group. Khorana score, previous use of anticoagulation or anti-platelet drugs did not differ between both groups, neither did the use of erythropoietin or steroids. Approximatively 90% of patients had a metastatic disease or a metastatic recurrence. No patient was treated with prior thoracic radiation. There were no significant differences between groups in terms of ECOG performance-status, stage, number of metastatic sites or presence of brain metastases. The tumor histology was an adenocarcinoma in 61.4% of the no-VTE group compared to 81.0% in the VTE group, but this difference was not statistically significant. In the no-VTE group, 44.3% of patients had a PD-L1 score <1%; 28.4% had a PD-L1 = 1-49% and 27.3% had a PD-L1≥50% compared to 38.1%; 23.8% and 38.1% respectively in the VTE-group but these differences were not statistically significant, nor was the difference observed in *KRAS* mutations (47.7% in the VTE group versus 31.8% in the no-VTE group). The mean duration of the first line CT-IO treatment was 6.4 months (maximum = 31.1 months).

**Table 1:** Characteristics of the population.

	Overall N=109	No-VTE N=88	VTE N=21	*p*-value
Demographic characteristics				
Women (%)	41 (37.6)	32 (36.4)	9 (42.9)	0.763
Age (years) median (Q1-Q3)	62.7 (56.8-66.9)	61.8 (56.8-68.4)	63.0 (57.4-65.4)	0.620
BMI median (Q1-Q3)	23.9 (21.0-26.9)	23.7 (21.0-26.9)	30.0 (25.0-40.0)	0.645
Smoking status (%)				0.634
Never	2 (1.9)	2 (2.3)	–	
Former	59 (54.6)	46 (52.9)	13 (61.9)	
Current	47 (43.5)	39 (44.8)	8 (38.1)	
Pack-year median (Q1-Q3)	40.0 (28.75-50.0)	40.0 (30.0-50.0)	30.0 (25.0-40.0)	0.258
Comorbidities (%)				
Chronic lung disease	35 (32.1)	30 (34.1)	5 (23.8)	0.518
Cardiovascular disease	54 (49.5)	43 (48.9)	11 (52.4)	0.963
Chronic renal failure	2 (1.8)	2 (2.3)	–	1.000
Liver failure	1 (0.9)	1 (1.1)	–	1.000
Auto-immune disorder	8 (7.3)	6 (6.8)	2 (9.5)	1.000
Prior VTE	1 (0.9)	1 (1.1)	–	1.000
Prior haemorrhage	7 (6.4)	4 (4.5)	3 (14.3)	0.254
Prior cancer	14 (12.8)	10 (11.4)	4 (19.0)	0.560
VTE risk factors (%)				
Recent surgery	8 (7.3)	5 (5.7)	3 (14.3)	0.372
Immobility	4 (3.7)	1 (1.1)	3 (14.3)	**0.025**
Anticoagulation				0.283
None	105 (96.3)	86 (97.7)	19 (90.5)
LMWH	2 (1.8)	1 (1.1)	1 (4.8)
DOAC	2 (1.8)	1 (1.1)	1 (4.8)
Antiplatelet drugs	27 (24.8)	23 (26.1)	4 (19.0)	0.693
Steroids	49 (45.0)	37 (42.0)	12 (57.1)	0.315
EPO	29 (27.1)	24 (27.9)	5 (23.8)	0.916
COC or HRT	1 (0.9)	0 (0.0)	1 (4.8)	0.434
VCC	107 (99.1)	86 (98.9)	21 (100.0)	1.000
Khorana Score				1.000
Intermediate risk	71 (75,5)	56 (74,6)	15 (79)
High risk	23 (24,5)	19 (25,4)	4 (21)
Cancer characteristics				
ECOG performance-status (%)				0.616
PS=0	60 (55.6)	49 (55.7)	11 (55.0)
PS=1	42 (38.9)	35 (39.8)	7 (35.0)
PS=2	6 (5.6)	4 (4.5)	2 (10.0)
Stage (%)				0.935
Stage III	10 (9.2)	8 (9.1)	2 (9.5)
Stage IV	81 (74.3)	66 (75.0)	15 (71.4)
Metastatic recurrence of stages I-II	18 (16.5)	14 (15.9)	4 (19.0)
Histology (%)				0.429
SCC	28 (25.7)	24 (27.3)	4 (19.0)
Adenocarcinoma	71 (65.1)	54 (61.4)	17 (81.0)
Other	10 (9.2)	10 (11.3)	–
Metastatic sites median (Q1-Q3)	3.0 (2.0-3.0)	2.0 (2.0-3.0)	3.0 (2.0-4.0)	0.200
Brain metastases (%)	29 (26.6)	24 (27.3)	5 (23.8)	0.962
PD-L1 (%)				0.619
< 1%	47 (43.1)	39 (44.3)	8 (38.1)
1-49%	30 (27.5)	25 (28.4)	5 (23.8)
≥ 50%	32 (29.4)	24 (27.3)	8 (38.1)
Genetic/molecular markers (%)				
*KRAS*	38 (34,9)	28 (31,8)	10 (47,7)	0.267
*BRAF*	2 (1,8)	2 (2,3)	–	1.000
*MET*	3 (2,7)	3 (3,4)	–	0.910

BMI, Body Mass Index; VTE, Venous Thromboembolism; LMWH, Low-weight molecular heparin; DOAC, Direct Oral Anticoagulant; EPO, Erythropoietin; COC, Combined oestrogen-progestin Oral Contraceptives; HRT, Hormone Replacement Therapy; VCC, Venous Central Catheter; SCC, Squamous-cell carcinoma.

The bold values denote statistical significance at P ≤ 0.05 level.

Concerning the use of anticoagulants in our cohort, a total of 29 patients (26.6%) were treated with anticoagulants: 3 patients (2.7%) were already under anticoagulant treatment for an atrial fibrillation before the cancer diagnosis (all three with DOACs), 21 (19.3%) received anticoagulation during the follow-up because of VTE (66.7% with LMWH and 28.6% with DOACs), and 6 (5.5%) received anticoagulation for a no-VTE indication. Besides, 27 patients (24.8%) had an antiplatelet treatment.

### 3.2 First VTE event

VTE occurred in 21 of 109 patients (19.3%) during a median follow-up of 13 months (IQR: 7.1 – 22.3 months). Among these, 13 (61.9%) were PE (associated or not to DVT); 2 (9.5%) were DVT alone; 3 (14.3%) were CRT, one (4.7%) was an extensive SVT and 2 (9.5%) were other site thrombosis. Four patients had PE associated with DVT at the same time (2 were inaugural and 2 during CT-IO treatment). Among them, only one patient had *in situ* PE with DVT.

**Figure 1:**
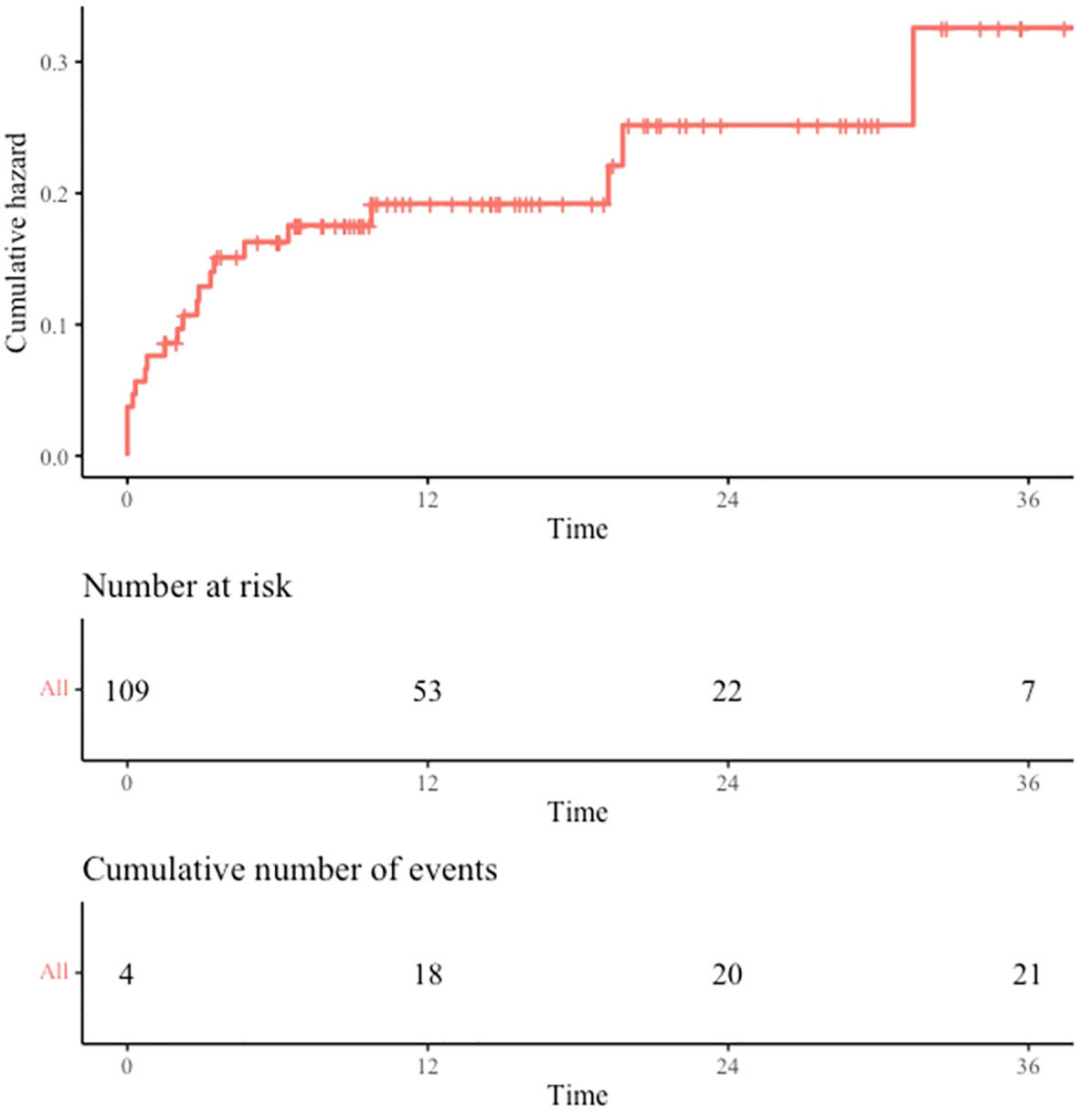
VTE incidence. Cumulative incidence of VTE from cancer diagnosis date. VTE occurring before cancer diagnosis are recorded at time 0. Time is expressed in months.

VTE was inaugural in 38.1% of cases, occurred during the first line CT-IO treatment in 38.1% of cases and after the first line treatment in 19% of cases. VTE incidence was especially high during the first 12 months after cancer diagnosis with a cumulative incidence at 3 months, 6 months and 12 months of 12.1% (95% CI: 5.7 - 18.1), 15.1% (95% CI: 8.0 - 21.6) and 17.5% (95% CI: 9.7 – 24.6) respectively (**Figure 1**). The median delay between the cancer diagnosis and the VTE occurrence was 62 days (IQR: 9-97 days).

Among the total VTE events, 14 (66.7%) were symptomatic while 7 (33.3%) were incidental.

The anticoagulant treatment for the first VTE event was LMWH in 66.7%, DOACs in 28.6% and one patient had an inferior vena cava filter. No patient was treated with VKAs in our cohort. One PE as first-VTE event (4.8%) occurred while the patient was under prior anticoagulant treatment (DOAC). No patient developed VTE while receiving bevacizumab or after.

### 3.3 VTE occurrence and PD-L1 expression

**Figure 2:**
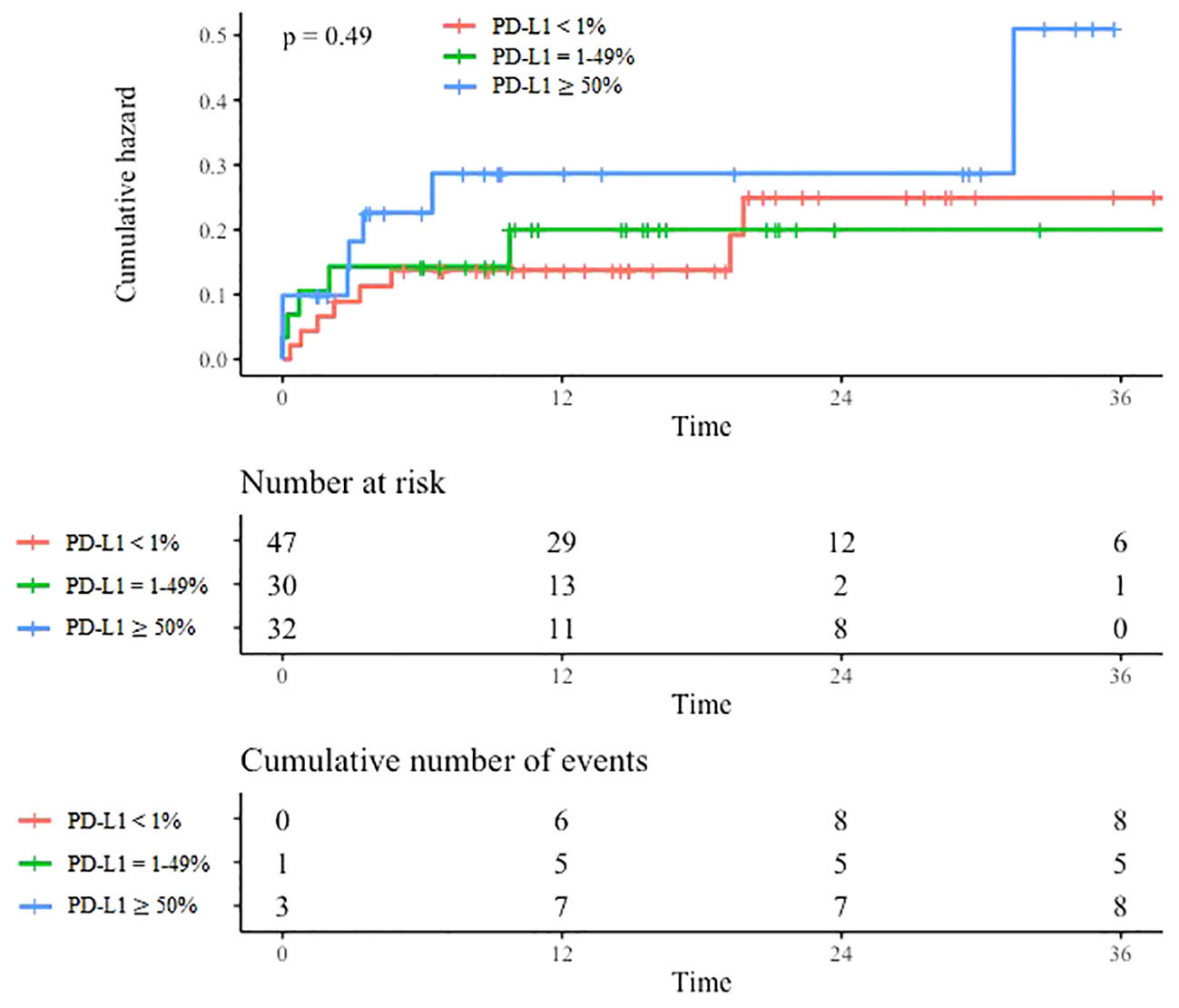
VTE incidence according to PD-L1 expression. Cumulative incidence of VTE according to PD-L1 expression. Time is expressed in months from cancer diagnosis.

In our study, VTE was not associated to PD-L1 tumor proportion score (**Figure 2**). No significant difference was observed when comparing VTE occurrence according to different PD-L1 scores (neither comparing PD-L1 <1% vs 1-49% vs ≥ 50%, nor comparing PD-L1 <1% vs ≥ 1% or PD-L1 <50% vs ≥ 50%). However, a trend emerged to higher PD-L1 scores in the VTE group, but this difference was not statistically significant.

### 3.4 Survival outcomes

**Figure 3:**
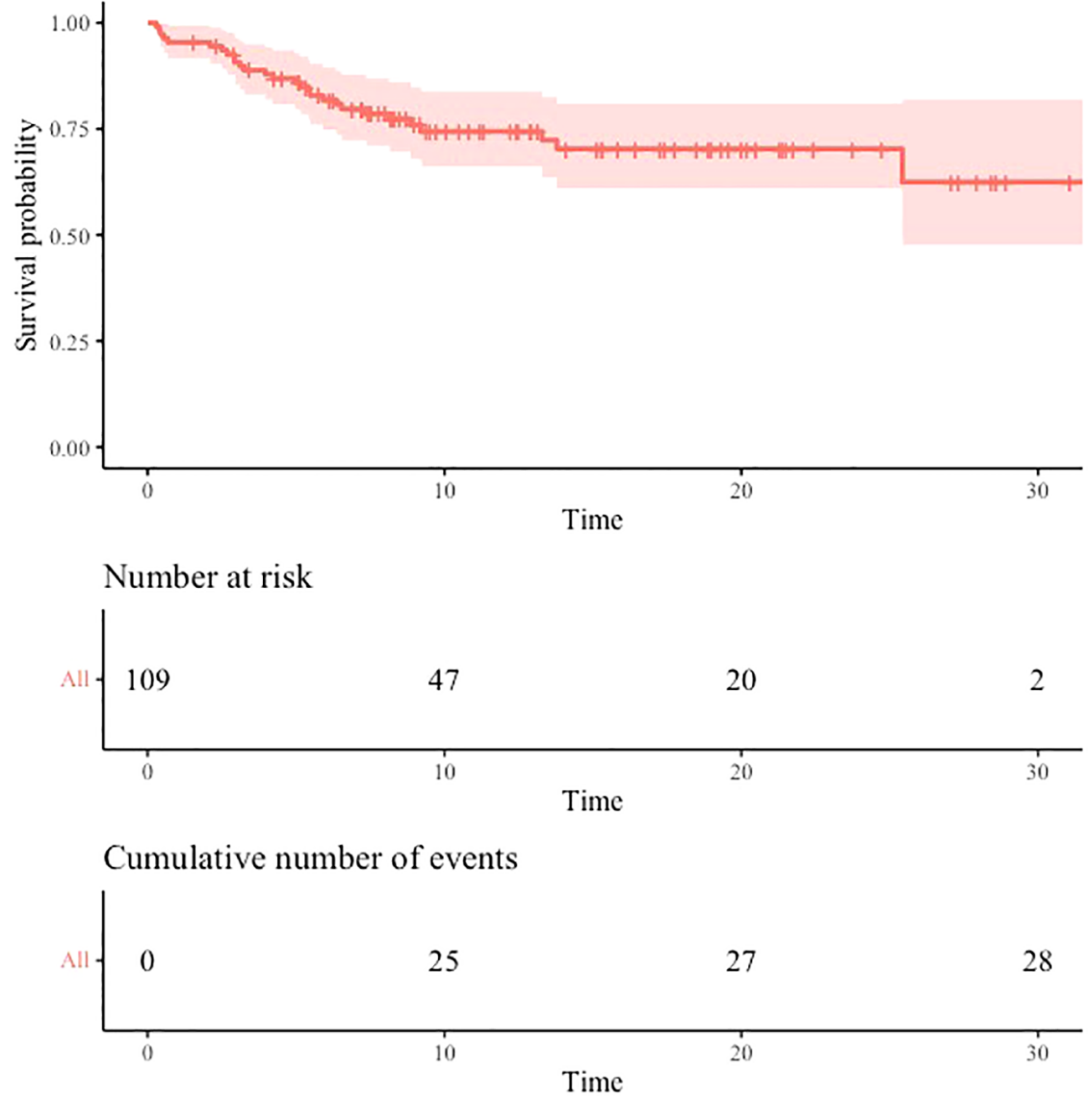
Overall survival. Estimated overall survival from start of first-line treatment. Time is expressed in months.

With a median follow-up of 13 months (IQR: 7.1 – 22.3 months), the estimated rates of OS at 6 months, 12 months and 24 months were respectively 81.9% (95% CI: 74.8-89.6), 74.4% (95% CI: 66.1-83.8) and 70.3% (95% CI: 61.0-81.1). Median OS was not reached (**Figure 3**). At the time of data cut-off, a total of 66 patients were still alive (60.6%) and 43 had died (39.4%).

### 3.5 Recurrent VTE

Four patients (19.0%) presented a recurrent VTE. They all occurred under anticoagulation therapy: three with standard doses of LMWH and one with DOAC. Three of the patients were women. None had a prior VTE history before the cancer diagnosis. Three patients had an intermediate Khorana score risk at diagnosis and one had a high risk. The latter was one of the two patients to present a second VTE recurrence. In all four cases, the first VTE event was inaugural. The VTE recurrence occurred 3 to 5 months after the first VTE event. No context of immobility or recent surgery was noted. All four patients had a metastatic adenocarcinoma and only one had brain metastases. Two had a PD-L1 score ≥ 50%, one had a PD-L1 score between 1-49% and one had a PD-L1<1%. Two harbored a *KRAS* mutation. Two of them presented a second VTE recurrence. Only one patient had atypical VTE associated with CRT at first and second recurrence. Management consisted in catheter removal and +25% increased dose of LMWH at first recurrence and no change at second recurrence because of clinical deterioration. All four patients died 1 to 4 months after the VTE recurrence and, on average, 6.3 months after the index VTE date. Three died due to cancer progression and one died due to a major bleeding complication. Recurrent VTE characteristics are summarized in **Table 2**.

**Table 2:** Recurrent VTE events.

	1^st^ VTE	1^st^ ACT	2^nd^ VTE	2^nd^ ACT	3^rd^ VTE	3^rd^ ACT	KS	PD-L1	Clinical evolution
**Patient 1** Male 62 years	PE + DVT	LMWH	Jugular vein thrombosis + CRT	+25% increased dose of LMWH	Upper vena cava thrombosis	No change	4	≥50%	Death due to disease progression
**Patient 2** Female 60 years	DVT alone	LMWH	PE + DVT	+25% increased dose of LMWH			2	≥50%	Death due to major bleeding
**Patient 3** Female 67 years	PE	DOAC	PE	standard dose of LMWH	CRT	+25% increased dose of LMWH	2	<1%	Death due to disease progression
**Patient 4** Female 55 years	SVT	LMWH (tinzaparin once daily)	PE	LMWH (enoxaparin twice a day)			2	1-49%	Death due to respiratory distress and disease progression

VTE, Venous thromboembolism; ACT, anticoagulant treatment; PE= Pulmonary embolism; DVT, Deep vein thrombosis; SVT, Superficial vein thrombosis; LMWH, Low-weight molecular heparin; DOAC, Direct Oral Anticoagulant; KS, Khorana score (0=low risk; 1 or 2 = intermediate risk; ≥ 3 = high risk); CRT, catheter-related thrombosis.

### 3.6 Bleeding complications under anticoagulant treatment

One patient (0.9%) presented a major bleeding complication in the form of a major hemoptysis leading to respiratory distress and patient’s death despite bronchial arterial embolization. The patient was a woman aged sixty who presented a metastatic adenocarcinoma with brain metastases and a PD-L1 score ≥ 50%. She did not suffer from hemostasis disorders nor from hepato-renal disorders (neither at the diagnosis nor at the time of the bleeding event) and had no prior bleeding history. She did not receive any anti-VEGF treatment. She was under supratherapeutic +25% LMWH dose for a recurrent VTE (according to French guidelines) when the bleeding complication occurred. Surprisingly, no minor bleeding events were reported in our study.

## 4 Discussion

Depending on the study, VTE incidence in lung cancer varies hugely ranging from 3% to more than 20% (17). In our cohort, the incidence of VTE (19.3%) was particularly high compared to previous studies in patients with NSCLC treated with chemotherapy but was consistent with more recent studies in patients treated with concomitant checkpoint inhibitors (18, 38). Indeed, Hill et al. recently reported a VTE incidence − considering only PE and DVT − of 9.9% at 6 months and 12.8% at 12 months in NSCLC patients treated with CT-IO. Former studies often recorded VTE events only in hospitalized patients or at the initial cancer diagnosis and did not consider VTE occurring in ambulatory patients or during the consecutive lines of systemic treatment. Besides, patients with prior VTE history or undergoing anticoagulation were often excluded. Most studies only recorded PE and DVT whereas we chose to include all VTE events, including CRT and other vein thrombosis (the rate of PE +/- DVT in our study was 13.8%) as well as incidental VTE events, as they all required a prolonged anticoagulation treatment and reflected a prothrombotic state.

Our analysis did not show a significant association between PD-L1 score and VTE occurrence. However, a trend emerged to higher PD-L1 scores in the VTE group and notably a higher VTE rate in patients with PD-L1 score ≥ 50% even if these results were not statistically significant. Other studies have previously shown an association between high PD-L1 scores and VTE (25, 27, 37). Absence of statistical significance in our study may be due to a lack of power because of its reduced size. On the other hand, the PD-L1≥ 50% patients that were included in our study may not be representative of all the PD-L1≥ 50% population as they probably had a more extended and aggressive disease than patients treated with single immunotherapy.

In our cohort, median follow-up achieved 13 months and surprisingly median OS was not reached. OS at 6 months was similar to the ones reported in clinical trials or other real-world studies of NSCLC treated with first-line CT-IO (39–41). However, OS at 12 and 24 months were higher than previously described despite our population was similar in terms of median age, sex, ECOG performance status, disease stage and PD-L1 distribution, and although we had a higher rate of brain metastases. Indeed, the latest results of phase III trials KEYNOTE-189 (pembrolizumab plus chemotherapy in metastatic nonsquamous NSCLC) and KEYNOTE-407 (pembrolizumab plus chemotherapy in metastatic SCC) reported median OS of 22.0 and 17.1 months respectively (22, 23). OS rates at 12 and 24 months were 69.8% and 45.7% in KEYNOTE-189 and 64.7% and 37.5% in KEYNOTE-407. A real-world study performed by Waterhouse et al. showed a median OS of 10.6 months in SCC and 12.0 months in nonsquamous NSCLC. OS at 12 and 24 months were 45.1% and 24.5% respectively in SCC and 49.9% and 32.5% in nonsquamous NSCLC. Highest OS in our study might be the consequence of the limited size of our cohort and the short follow-up resulting in less precise estimations at 12 and 24 months with wider 95% CI. Survival analyses according to PD-L1 expression were not performed as it was not the aim of this study and as prior studies already showed that high PD-L1 scores were associated with higher OS (39–41). Survival analyses according to VTE occurrence were not performed as this would have required more sophisticated statistical analysis considering the competing risk of death and as it is well recognized that cancer patients experiencing VTE have a shorter survival.

We found a higher VTE recurrence rate (19%) than reported in previous trials comparing DOACs versus LMWH for the treatment of cancer-associated VTE (12–15, 42) but similar to other real-world studies (7, 16). Only one of the four patients presenting a VTE recurrence had a high Khorana score risk highlighting the poor prognostic value of this indicator. We observed a higher proportion of PD-L1 ≥ 1% among patients presenting a VTE recurrence but, because of the limited size of our study population, no conclusion with a statistical significance can be driven by this observation. VTE recurrences occurred in the first months after the first VTE event. Patients experiencing a VTE recurrence died shortly after, highlighting the impact of VTE recurrences on survival and suggesting an aggressive underlying disease.

Only one patient (0.9%) presented a major bleeding complication while under anticoagulant treatment. No clinically relevant non-major (CRNM) bleeding event was noted, probably because these events were unreported in the medical records. Besides, we did not consider minor hemoptysis as CRNM bleedings since it is a sign of the disease itself rather than a clinically relevant complication. We did not find any study reporting bleeding events among lung cancer patients; we only found studies evaluating the efficacy and safety of anticoagulants for VTE in lung cancer patients (42). The main limitations of our study were its retrospective design and the reduced size of the cohort as well as a limited follow-up duration. However, ours is one of the first studies assessing VTE incidence in a real-world cohort of NSCLC patients treated with CT-IO as first-line therapy and evaluating the role of PD-L1 as a possible predictor marker of VTE. Our study brings new data on this frequent and serious complication in NSCLC in the era of chemo-immunotherapy associations. Further studies with a prospective design and more patients are needed to assess more accurately the risk of VTE associated to different anticancer therapies and tumor markers, especially if high PD-L1 expression might be associated with VTE.

## 5 Conclusion

VTE is a common and serious complication in advanced NSCLC patients treated with first-line concomitant chemo-immunotherapy, often inaugural or occurring in the first months after cancer diagnosis. The incidence of VTE achieved 19.3% in our study. PD-L1 did not appear to be statistically associated with VTE occurrence. We recorded high VTE recurrence rates despite anticoagulant treatment. Further investigations are needed to determine if high PD-L1 expression is associated with VTE.

## Data availability statement

The original contributions presented in the study are included in the article/supplementary material. Further inquiries can be directed to the corresponding author.

## Ethics statement

The studies involving humans were approved by Institutional Review Board of the French learned society for respiratory medicine – “Société de Pneumologie de Langue Française” – (reference CEPRO 2022-041). The studies were conducted in accordance with the local legislation and institutional requirements. The participants provided their written informed consent to participate in this study.

## Author contributions

Category 1: Conception and design of study: LA, MG, CO, RD. Acquisition of data: LA.Analysis and/or interpretation of data: LA, MG, CO. Category 2: Drafting the manuscript: LA, MG, CO. Revising the manuscript critically for important intellectual content: All authors. Category 3: Approval of the version of the manuscript to be published (the names of all authors must be listed): LA, MG, CO, RD, CT, FC.
